# Composite Nitride Nanoceramics in the System Titanium Nitride (TiN)-Aluminum Nitride (AlN) through High Pressure and High Temperature Sintering of Synthesis-Mixed Nanocrystalline Powders †

**DOI:** 10.3390/ma14030588

**Published:** 2021-01-27

**Authors:** Mariusz Drygaś, Katarzyna Lejda, Jerzy F. Janik, Bogdan Musielak, Stanisław Gierlotka, Svitlana Stelmakh, Bogdan Pałosz

**Affiliations:** 1 Department of Fuels Technology, Faculty of Energy and Fuels, AGH University of Science and Technology, Mickiewicza 30, 30-059 Krakow, Poland; madrygas@agh.edu.pl (M.D.); kkapusta@agh.edu.pl (K.L.); 2 Faculty of Chemistry, Jagiellonian University, Gronostajowa 2, 30-387 Krakow, Poland; musielak@chemia.uj.edu.pl; 3 Institute of High Pressure Physics, Polish Academy of Sciences, Sokołowska 29/37, 01-142 Warszawa, Poland; stanislaw.gierlotka@unipress.waw.pl (S.G.); svrit@mail.unipress.waw.pl (S.S.); bogdan.palosz@unipress.waw.pl (B.P.)

**Keywords:** nitrides, nanocrystalline composites, sintering, nanoceramics, Vicker’s hardness

## Abstract

Presented is a study on the original preparation of individual and in situ intimately mixed composite nanocrystalline powders in the titanium nitride-aluminum nitride system, Ti:Al = 1:1 (at.), which were used in high pressure (7.7 GPa) and high temperature (650 and 1200 °C) sintering with no binding additives for diverse individual and composite nanoceramics. First, variations in precursor processing pathways and final nitridation temperatures, 800 and 1100 °C, afforded a pool of mixed in the nanosized regime cubic TiN (c-TiN) and hexagonal AlN (h-AlN) composite nanopowders both with varying average crystallite sizes. Second, the sintering temperatures were selected either to preserve initial powder nanocrystallinity (650 °C was lower than both nitridation temperatures) or promote crystal growth and recrystallization (1200 °C was higher than both nitridation temperatures). Potential equilibration towards bimetallic compounds upon solution mixing of the organometallic precursors to nanopowders, monomeric Ti[N(CH_3_)_2_]_4_ and dimeric {Al[N(CH_3_)_2_]_3_}_2_, was studied with ^1^H and ^13^C NMR in C_6_D_6_ solution. The powders and nanoceramics, both of the composites and individual nitrides, were characterized if applicable by powder XRD, FT-IR, SEM/EDX, Vicker’s hardness, and helium density. The Vicker’s hardness tests confirmed many of the composite and individual nanoceramics having high hardnesses comparable with those of the reference h-AlN and c-TiN ceramics. This is despite extended phase segregation and, frequently, closed microsized pore formation linked mainly to the AlN component. No evidence was found for metastable alloying of the two crystallographically different nitrides under the applied synthesis and sintering conditions. The high pressure and high temperature sintering of the individual and in situ synthesis-mixed composite nanopowders of TiN-AlN was demonstrated to yield robust nanoceramics.

## 1. Introduction

Artificial main group metal/metalloid and transition metal nitride nanostructured thin films and powders are unparalleled engineering materials with an ever-increasing impact on technology [1,2,3,4]. All this is possible due to the materials’ unique properties and, generally, robust behavior towards oxidation even at elevated temperatures. The notable exception includes high surface area nanopowders that are by their very nature distinctly more than crystallized thin films prone to deterioration in the air atmosphere. Significantly, a group of direct semiconductors, including main group nitrides GaN, InN, and their solid solutions with AlN, have become crucial in modern electronics for the development of blue-to-green light emitting diode (LED) and laser sources and, eventually, efficient phosphors for ever more affordable common-day white LED applications [5,6,7]. In another spectrum, traditional ceramic applications that mainly utilize the materials’ mechanical and thermal properties are characteristic for many transition metal nitrides, including TiN, ZrN, and WN but incorporating also such main group compounds such as BN, AlN, and Si_3_N_4_ (each of which already plays a significant role in various technologies) [8,9,10,11,12]. Many emerging applications of simple and complex (alloyed and composite) nitrides exploit the synergy advantages of coexisting electronic and mechanical/thermal properties [1,2,3,4,13]. In this regard, the subject of this study is one of the important composite nitride systems made of AlN and TiN, ceramics of which may have the combined advantages of the insulating and heat-conducting properties of AlN and the low electrical resistivity and mechanically robust nature of TiN, yielding an unusual set of functional features. The standard reference nitrides’ properties have usually been derived for monocrystals, coarse polycrystalline powders or crystalline films. These properties can be significantly different for nanostructured materials forms including nanocrystalline powders, where crystallite size-related effects and increased specific surface area come to play an essential role. Excluding the latter property, this is also true for the sintered materials forms, especially if prepared as nanoceramics made of nanosized crystalline components.

Main group aluminum nitride is considered an electrical insulator with a direct bandgap of 6.2 eV, which is prevailingly synthesized as the stable stoichiometric hexagonal, wurzite-type variety h-AlN [5,6,7], although there have been observations of rather unusual cubic polytype formation as well [14]. AlN’s outstanding property is high thermal conductivity, which ranges, typically, from 170–200 W/m·K for polycrystalline powders to 320 W/m·K for monocrystals, and is twice that of silicon, which is remarkably high among insulators. It shows negative electron affinity that well compares to that of diamond. Moreover, it has high thermal stability and advantageous mechanical toughness. AlN is commercially available as microcrystalline powders made mostly by carbothermal reduction of alumina Al_2_O_3_ or direct nitridation of metal aluminum powders, although there are several specific methods that use selected aluminum inorganic and organometallic precursors for the preparation of nanosized powders [1,15,16,17,18].

Transition group titanium nitride, which tolerates some nitrogen deficiency, is stable in ambient conditions as a cubic polytype (rock salt c-TiN or c-TiN_1−x_) [19,20,21]. It shows a very high metallic-type electrical conductivity and has outstanding mechanical and refractory characteristics. This extremely hard nitride of gold color (like gold it reflects infrared radiation) is often considered for ceramic/protective layers on cutting tools including medical devices and bio-compatible implants but also for jewelry for decorative purposes. TiN can be efficiently prepared by nitridation of titanium metal powders or titanium chloride with nitrogen/ammonia and by carbothermal reduction-nitridation of various titanium oxygen-bearing precursors. Many of these syntheses have been directed towards thin film nanostructures [22,23,24] and nanocrystalline powders [25,26,27].

In the ternary element system Ti-Al-N, the thermodynamically stable assembly at room temperature is made of a mixture of h-AlN and c-TiN since the two crystallographically different nitrides do not alloy to form a detectable solid solution at ambient conditions [28,29,30]. The latter, for instance, takes place for any of the binary nitride systems of AlN/GaN/InN in the entire composition range [5]. In addition to the two binary metal nitrides, three ternary compounds, namely, Ti_2_AIN, Ti_3_A1N, and Ti_3_A1_2_N_2_, are found in the Ti-A1-N system, which are stable only at elevated temperatures. This has to be confronted with the established, for nearly three decades, various technologies for large scale preparations of the very hard and oxidation resistant coatings described as made of cubic Ti_1−x_Al_x_N, i.e., a structure formed by substituting some titanium centers with aluminum ones in the reference cubic titanium nitride. Such a metastable phase has been prepared mostly in the form of thin films via various gas phase deposition techniques at relatively high temperatures to wide utilization at ambient to mild conditions [31,32,33,34]; these films decompose to the stable polytypes of h-AlN and c-TiN at suitably high temperatures in the order of 800–1000 °C and higher [35,36]. In rare cases, the “phase of Ti_1−x_Al_x_N” has appeared up-front to be an intimate mixture of c-TiN and amorphous AlN domains [35]. Otherwise, the hard and resistant coatings were unusually shown to have a metastable hexagonal Ti_1−x_Al_x_N structure [37]. In conclusion, even under elevated temperature conditions, the powder forms of h-AlN and c-TiN are not expected to form solid solutions but rather to coexist as a heterogeneous composite system. However, based on what is known, it is unclear what could be a propensity for the metastable solid solution formation of cubic Ti_1−x_Al_x_N upon a concerted action of high temperatures and high pressures as often applied in no-additive sintering.

Powder sintering with no binding aids has been frequently reported for the system Ti-Al-N to make mechanically robust bulk material forms. Sintered compacts of TiN and AlN (including some adventitious Al_2_O_3_) with high densities and significant Vicker’s hardness were prepared by, first, mechanical alloying of commercial powders of AlN, Ti, and ammonium carbonate and, second, application of either hot pressing (400 MPa, 1473 K, N_2_ atmosphere) or spark plasma sintering (50 MPa, 1523 K, vacuum) [38]. Hot pressing of various TiN and AlN powder mixtures afforded a pool of compact ceramics with high Vicker’s hardness and controlled wear resistance [39], whereas the electric spark-assisted process was applied for such powder mixtures to make wear-resistant TiN-AlN coatings [40]. Commercial microcrystalline powders of TiN and AlN were, first, high-energy ball milled and, second, consolidated with the pulse current activated sintering process to yield TiN-AlN ceramics with near theoretical densities and Vicker’s hardness sometimes exceeding the hardness of monolithic TiN [41]. Consolidation of the high-energy ball-milled commercial TiN + 5% AlN powders was demonstrated by application of microwaves (2.45 GHz) at temperatures up to 1550 °C [42]. Composite TiN-AlN ceramics with components in ratios 1:3, 1:1, and 3:1 were produced by, first, mechanical alloying of the commercially available microcrystalline powders and, second, hot isostatic pressing (up to 1900 °C, 220 MPa, 90 min); the ceramics consisted of a mixture of h-AlN and c-TiN, the latter component proposed to contain some “dissolved” AlN [43]. Ultradispersed (dispersion method not reported) powders of AlN and TiN were mill-mixed, hot pressed at 220 MPa, and sintered at 800–1900 °C and 100 KPa pressure yielding composite compacts with 12–15% porosity; an inhibiting particle growth behavior was noted and ascribed to the lack of contacts among alike particles in the mixture [44]. Commercial microcrystalline powders of c-TiN and h-AlN (variable amounts of 15 or 25 vol.% h-AlN were added to increase mechanical properties and reduce porosity) were mixed/alloyed by dry-ball milling (600 rpm, 3–6 h) before compaction by spark plasma sintering (1600–1700 °C) to afford hard compacts with the Vicker’s hardness of 19–20 GPa and near theoretical density; in addition to c-TiN and h-AlN, some quantities of an oxygen-bearing spinel phase AlON (5–8 vol.%) were detected [45]. Spark plasma sintering was also reported for commercial microcrystalline powders of TiN and AlN. These were, first, milled for up to 100 h to yield, in some cases, the proposed solid solution of cubic (Ti,Al)N and, second, consolidated/spark plasma-sintered at 1273–1423 K to form very hard compacts that appeared to be composites of c-TiN and amorphous or hexagonal AlN; this observation was indicative of the decomposition of the initial metastable cubic (Ti,Al)N phase under the applied compacting conditions [46]. Nitrogen-deficient titanium nitride TiN_1−x_ was first made by mechanical alloying and, subsequently, mixed with commercial microcrystalline AlN (up to 30 vol.%) to be cold pressed and sintered under high pressure and high temperature conditions (5 GPa, 1400–1600 °C, 15 min) to result in nano-AlN-TiN-TiN_1−x_ ceramics with unusual epitaxial interfaces and very high Vicker’s hardness [47]. Commercial h-AlN (97%) and c-TiN (3%) microcrystalline mixtures were subjected to high pressure and high temperature sintering (12 GPa, 1472–1772 K, 3 min) and afforded an unexpected ceramics containing, in addition to h-AlN (94%) and c-TiN (3%), some unstable cubic c-AlN polytype (3%), likely stabilized by some incorporated Ti-centers [48].

As can be deduced from this concise overview, both nitrides, individually or as the metastable ternary phase and/or composites, are mostly utilized as thin nanostructured films (most often made via gas phase deposition) or bulk ceramics made, in vast majority of cases, from commercial microcrystalline powders. In this regard, the nanopowder materials form appears to offer significant advantages through increased surface-related reactivities and specific dependencies of many properties on crystallite dimensions in this particle size regime. One has to realize, however, that elaboration of reproducible synthesis routes to nanopowders, both individual and composite, with controlled grain size distribution characteristics and surface properties is a non-trivial research challenge. All this is also true for subsequent nanopowder conversions to ceramic functional forms, mechanically compact and machinable materials, that could potentially serve in various applications as substitutes for the unavailable or difficult to make and expensive monocrystals.

We recently reported a related study on the preparation of mixed metal nitride nanopowders via transamination-deamination chemistry applied to selected metal organoamide derivatives in the system AlN-GaN, which was followed by no-additive high pressure and high temperature sintering of the nanopowders [49]. In that case, in addition to the binary nitride composites AlN-GaN, the nitrides stable solid solution Al_x_Ga_1-x_N, 0 < x < 1, was already partially formed (or, to phrase it alternatively—the nitrides alloyed) in the nanopowder preparation stage and, under suitable conditions, continued to form during the subsequent high pressure and high temperature sintering (7.7 GPa, 1000 °C). In particular, in one of the experimental routes, novel nanoceramics made exclusively of the solid solution Al_0.5_Ga_0.5_N were prepared. The chemical foundations for such an outcome are based (i) on the specific reactivity of the organometallic precursors and (ii) on the actual thermodynamic stability of Al_x_Ga_1−x_N. The latter is linked to the similar chemical bonding characteristics but also to the same stable hexagonal polytype of both individual nitrides. The former aspect, as previously reported by some of us [18], is based on the observation that in mixed hexane solutions of the dimeric Al- and Ga-tris(dimethylamides), {M[N(CH_3_)_2_]_3_}_2_, M = Al, Ga, Al:Ga = 1:1, no detectable formation of a mixed bimetallic Al/Ga-tris(dimethylamide) dimer takes place at room temperature, whereas under reflux conditions the mixed dimer [(CH_3_)_2_N]_2_Al-[*μ*-N(CH_3_)_2_]_2_Ga[N(CH_3_)_2_]_2_ amounts to about 50%. Such a system of intimately mixed monometallic and bimetallic dimers, when subjected to further ammonolysis reactions with ammonia, affords a solid amide-imide precursor already containing -N-Al-N-Ga-N- linkages that favor the Al_x_Ga_1−x_N solid solution formation upon final nitridation at increased temperatures. And indeed, the nitrides solid solution was shown to be partially formed in specific cases during the mixed nitride nanopowder synthesis stage. Suitably high temperatures during sintering, if required, promoted the alloying reactions of the available/remaining AlN and GaN nitrides and resulted in increased quantities of the solid solution up to pure Al_0.5_Ga_0.5_N.

In this regard, such a course of events seems rather improbable in the relevant TiN-AlN system since none of the two chemical foundations discussed above appears to be true. Namely, (i) the monomeric Ti-tetra(dimethylamide) and dimeric Al-tris(dimethylamide), which are to be used as the initial precursors, are not likely to form the bimetallic -N-Ti-N-Al-N- linkages, and (ii) the eventual end-up solid solution Ti_1−x_Al_x_N is not thermodynamically stable. Though unlikely, the formation of some metastable Ti_1−x_Al_x_N cannot be a priori excluded, especially, in the sintering stage given the then applied extreme pressure and temperature conditions.

Herein reported is a study on the application of two different processing variations of the binary organometallic precursor system in the original in situ preparation of the intimately mixed composite nanopowders of TiN-AlN. In particular, the ammonolysis of the solution-mixed organometallic precursors followed by nitridation of the resultant metals’ amide-imide precursors at selected temperatures yielded a diverse pool of the reaction-mixed composite nitrides nanopowders. This was followed by the nanopowders high pressure and high temperature sintering towards robust composite TiN-AlN nanoceramics. In parallel experiments, similar syntheses were carried out for the individual organometallic precursors to result in pure nanocrystalline nitrides of TiN and AlN that were then sintered towards the pure nitride nanoceramics for reference purposes.

## 2. Experimental

### 2.1. Preparation of Metal Amide-Imide Precursors in the Mixed Bimetallic Tetrakis/tris(dimethylamide) System │Ti[N(CH_3_)_2_]_4_/{Al[N(CH_3_)_2_]_3_}_2_│/NH_3_, Atomic Ratio Ti:Al = 1:1 and Individual Reference Systems of │Ti[N(CH_3_)_2_]_3_}_2_│/NH_3_ and │{Al[N(CH_3_)_2_]_3_}_2_│/NH_3_

#### 2.1.1. Preparation of Mixed Precursor 1 via Reaction at Room Temperature (RT) and Short Equilibration Time

Samples of Ti[N(CH_3_)_2_]_4_, 7.18 g (32.0 mmol of monomer), and {Al[N(CH_3_)_2_]_3_}_2_, 5.10 g (16.0 mmol of dimer), were made according to the published procedures, dissolved together in 60 mL of dry hexane, and stirred at RT for 10 min. This was equivalent to a negligible mixed-metal dimer formation in the similar but reactive Al/Ga-amide system [18]. Upon hexane evaporation, liquid NH_3_ (60 mL) was transferred onto the solid, and the mixture was stirred under reflux at ca. −33 °C for 2 h, followed by a 2-h NH_3_ boil-off at this temperature. The resulting white solid was evacuated at RT for 0.5 h, thus affording polymeric Ti-imide/Al-amide-imide Precursor 1.

#### 2.1.2. Preparation of Mixed Precursor 2 via 3-h Reflux in Hexane Solution

Samples of Ti[N(CH_3_)_2_]_4_, 8.97 g (40.0 mmol of monomer), and {Al[N(CH_3_)_2_]_3_}_2_, 6.38 g (20.0 mmol of dimer), were made as previously described, dissolved together in 60 mL of hexane, and refluxed for 3 h. In the similar but reactive Al/Ga-amide system, this was equivalent to ca. 50% bimetallic dimer {Al/Ga[N(CH_3_)_2_]_3_}_2_ [18]. The subsequent work-up was identical as above and afforded polymeric Ti-imide/Al-amide-imide Precursor 2.

#### 2.1.3. Preparation of Reference Pure Ti- and Al-Precursors

The reference Ti-imide [50,51] and Al-amide-imide [18,52] precursors for individual nitrides TiN and AlN were made, respectively, from liquid titanium tetrakis(dimethylamide) and solid aluminum tris(dimethylamide) by ammonolysis of the samples in liquid ammonia under the same conditions as applied for mixed bimetallic Precursors 1 and 2.

### 2.2. Nitridation Towards Nanopowders

Mixed bimetallic Precursors 1 and 2 as well as the individual precursors of Ti-imide and Al-amide-imide were used in pyrolysis experiments. The experiments were performed in an alumina heated tube under a flow of NH_3_, 0.2 L/min, for 4 h at two selected temperatures, 800 and 1100 °C, for each precursor loaded in a closed-end alumina boat. The products, each in the amount of ca. 1.3–1.5 g, were dark brown or greyish free-flowing powders that were stored in a glove-box for analytical determinations. The samples for sintering experiments were loaded to glass ampoules that were sealed under vacuum and opened directly before sintering.

### 2.3. High Pressure and High Temperature Sintering

Upon ampoule opening, the powders were removed and briefly handled in air prior to the high pressure and high temperature sintering using the methodology worked out earlier by some of us [49,53,54]. Specifically, the powders were sintered for 3 min in a high pressure torroid cell at 650 and 1200 °C under the pressure of 7.7 GPa, to yield dark brown or dark grey ceramic pellets, D = 4 mm, of thickness ca. 2–3 mm. For Vicker’s hardness determinations on a pellet, one of its sides was polished. For other measurements, the pellets were coarsely crushed/ground in an agate mortar and used as such.

**Figure 1:**
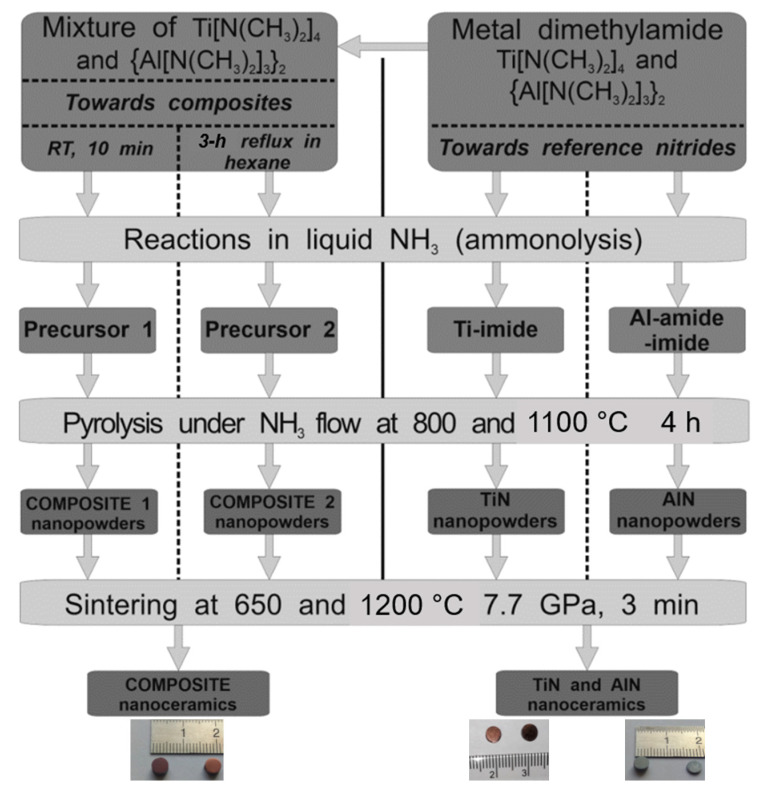
Schematics of experimental steps in anaerobic synthesis and subsequent high pressure and high temperature sintering of composite and individual nanopowders in the system TiN-AlN. Snapshots of typical pellets/nanoceramics are shown on the bottom as follows: (**left**)—brown composite pellets; (**right**)—golden-brown TiN pellets and graphite-grey AlN pellets.

The major steps in the nitrides synthesis and sintering are shown in Figure 1.

### 2.4. Nitride/Ceramics Sample Labeling

The powders prepared from molecular Precursors 1 and 2 were labeled, respectively, Composites 1 and 2 with an additional reference to nitridation temperature. For example, for Precursor 1, after nitridation at 800 and 1100 °C, two products were obtained, i.e., Composite 1_800 and Composite 1_1100, respectively, etc. The powders made from pure metal precursors were the individual nitrides of TiN and AlN and were labeled accordingly. For example, for Ti-imide pyrolyzed at 800 and 1100 °C, the two powder products were, respectively, TiN_800 and TiN_1100, etc. The sintered ceramics had names of the related nitride powders with suitable postfixes for sintering temperature, e.g., Composite 1_800_sint_650 or TiN_1100_sint_1200, etc.

### 2.5. Characterization

Proton (^1^H) and carbon (^13^C) nuclear magnetic resonance (NMR) spectra were acquired using a Bruker Avance III 600 MHz spectrometer (Billerica, MA, USA) equipped with the nitrogen cryo-probe head at 300 K. Spectra were recorded in C_6_D_6_ solutions contained in sealed 5 mm glass tubes, and the ^1^H and ^13^C chemical shifts were referenced to the residual solvent signals and adjusted to TMS at 0 ppm. The spectra for Precursor 1 were run as soon as possible after the RT, 10 min-equilibration followed by solution work-up and sample preparation in C_6_D_6_ (approx. 3 h past equilibration matching the timespan of the next step, i.e., transamination reactions in liquid NH_3_). Accordingly, the spectra for Precursor 2 were acquired after the hexane reflux equilibration. Powder X-ray diffraction (XRD) determinations were done for all nitride products (powders and sintered ceramics) by Empyrean PANalytical (Malvern, UK), Cu Kα source, 2θ = 20–80°. Due to small crystallite sizes, the peaks were broadened and overlapped in many cases, resulting in decreased accuracies of crystallite cell parameter determinations down to ca. 0.01 Å. For the purpose of this study, average crystallite sizes were evaluated from Scherrer’s equation applying the standard Rietveld refinement method. Fourier transform infrared (FT-IR) determinations were carried out on a Nicolet 380 spectrometer (Waltham, MA, USA) in KBr pellets made in dry-box. Scanning electron microscopy (SEM) standard imaging data were acquired for carbon-coated samples with a Hitachi Model S-4700 microscope (Tokyo, Japan), whereas SEM with energy dispersive X-ray (EDX) analysis and element mapping were done for the polished side of a pellet on Zeiss Supra Leo 1530 (Oberkochen, Germany). Helium densities were obtained by Micromeritics AccuPyc 1340 pycnometer (Norcross, GA, USA).

The Vicker’s hardness (H_v_) tests were performed on micro-hardness tester FutureTech FM-700 (Kanagawa, Japan) with a 100 and 300 gf (gram-force) load on a polished pellet surface, 10 s, and hardness expressed in GPa. The data sets for the two loads were comparable and the more consistent set of the H_v_ values recorded for 300 gf was selected for discussion. Five to ten measurements were carried out for each pellet to calculate an average H_v_ value and its standard deviation.

## 3. Results and Discussion

### 3.1. Nitride Synthesis

In the reference system of dimeric Al-tris(dimethylamide) plus dimeric Ga-tris(dimethylamide) refluxed in hexane, as outlined above, the presence of some mixed dimeric Al/Ga-tris(dimethylamide) was confirmed and shown to be linked to Al_x_Ga_1−x_N solid solution formation upon further ammonolysis and nitridation [18,49]. In this regard, in the monometallic case of M = Al or Ga, a simplified sequence of reactions of interest includes the (i) ambient temperature transamination of dimeric M-amide + ammonia → polymeric M-amide-imide + dimethylamine and (ii) pyrolytical deamination/nitridation of M-amide-imide → M-nitride + ammonia. The course of the reactions is similar for the bimetallic system, but the transamination in the system refluxed in hexane is thought to also lead to some mixed M-amide-imide species (M = Al/Ga) setting preferences in the subsequent nitridation stage for the metal nitrides solid solution formation, as is indeed observed. In the actual bimetallic system of monomeric Ti-tetrakis(dimethylamide) and dimeric Al-tris(dimethylamide), the formation of the bimetallic Ti/Al-tetrakis/tris(dimethylamide) seems unlikely just for steric factors, although it cannot be up-front excluded. That is the reason for applying two processing pathways via the Precursor 1 and Precursor 2 routes (Figure 1), with the latter possibly favoring mixed Ti/Al-species formation, hypothetically towards mixed-metal cubic Ti_1−x_Al_x_N.

**Figure 2:**
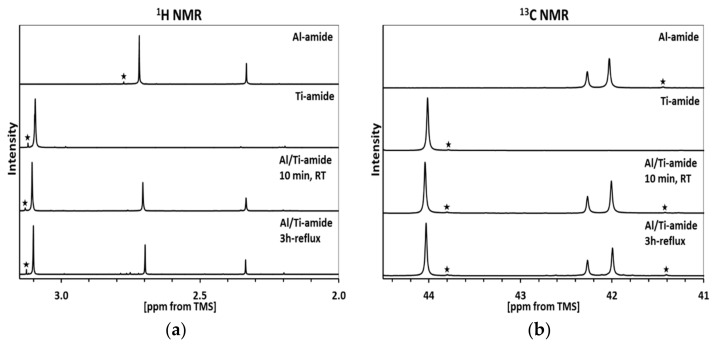
^1^H (**a**) and ^13^C (**b**) nuclear magnetic resonance (NMR) spectra in C_6_D_6_ of individual M-dimethylamides (M = Al or Ti) and bimetallic mixtures of Al/Ti-dimethylamides for two initial precursor processing routes/equilibration pathways. Small intensity peaks with asterisk indicate very low contamination levels of a compound’s decomposition products formed during their final purification by vacuum sublimation/distillation.

In order to check the possibility of the mixed Ti/Al-dimethylamide species formation, the ^1^H and ^13^C NMR spectra were collected after the equilibration steps of the Precursors 1 and 2 routes (Figure 2).

The spectra for pure Al-tris(dimethylamide) (Figure 2, top) confirm its dimeric character in the solution with 2 bridging and 4 terminal –N(CH_3_)_2_ groups to yield two non-equivalent proton (2.34 and 2.72 ppm) and carbon (42.3 and 42.0 ppm) sites, both with intensity ratio 1:2 [18]. On the other hand, the spectra for pure Ti-tetrakis(dimethylamide) (Figure 2, second from top) are consistent with its monomeric character resulting in the single ^1^H (3.09 ppm) [55] and ^13^C (44.1 ppm) signals. For the differently equilibrated mixtures (Figure 2, bottom and second from bottom), both the proton and carbon spectra are essentially unchanged; the small variations of the order of ca. 0.01–0.02 ppm for the proton spectra and less than 0.1 ppm for the carbon spectra are likely due to concentration-dependent effects and insignificant from the point of view of major structure changes. In essence, both solution mixtures appear on the NMR time scale to just be made of the monomeric Ti-tetrakis(dimethylamide) and dimeric Al-tris(dimethylamide). Therefore, this stage of precursor processing should be rather neutral for a potential formation of the metastable bimetallic Ti_1−x_A_x_N along the nitridation pathways to Composites 1 and 2.

**Figure 3:**
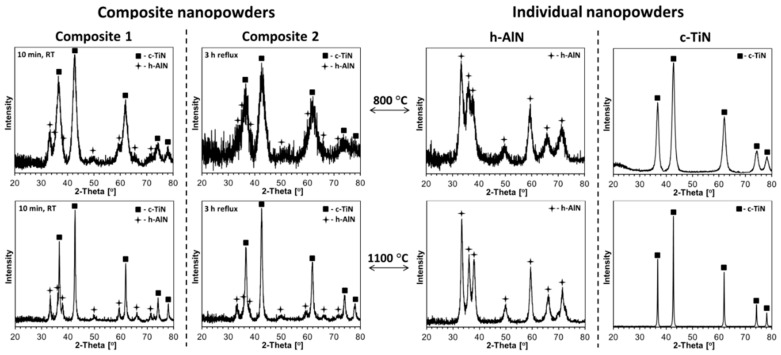
X-ray diffraction (XRD) patterns of composite (**left panel**) and individual (**right panel**) nitride nanopowders prepared at 800 and 1100 °C in the system TiN-AlN.

The XRD patterns for the nanopowders from two composite nitride processing routes (Composites 1 and 2) and reference pure nitrides (h-AlN and c-TiN), all prepared at both 800 and 1100 °C, are presented in Figure 3. The calculated cell parameters for h-AlN and c-TiN in composites and in individual nitrides as well as estimated average crystallite sizes are included in Table 1.

The data for the composite nanopowders show all of them to be mixtures of c-TiN and h-AlN after the 800 and 1100 °C nitridation. There is no clear evidence for a presence of the potential metastable cubic Ti_1−x_A_x_N with significant quantities of aluminum in the lattice, which would have then caused much smaller values of the *a* constant in the cubic phase than the observed 4.23–4.24 Å, the latter typical for c-TiN [19,20,21]. The crystallite cell parameters for the 800 and 1100 °C pairs of products, i.e., for Composites 1 and 2, differ slightly. This is especially true for the h-AlN component, wherein the *a* and *c* lattice constants for the 1100 °C-products approach the literature values for h-AlN of a = 3.11 Å and c = 4.99–5.00 Å [5,6,7], while for the 800 °C-products, they are noticeably different although still following the temperature-related trend observed for the relevant pair of the reference pure AlN powders (Table 1). These XRD data agree well with our general observations of cell parameter dependencies on average crystallite size in the low nanosized range. Moreover, the lattice constants for the 800 °C-powder of Composite 2 are likely to be of very low accuracy due to severe broadness of the AlN peaks that are overlapped by the stronger/narrower peaks for TiN (Figure 3, left panel, top right pattern). In conclusion, there are no crystallographic indications of the bimetallic nitride phase in either Composite 1 or Composite 2 at the applied nitridation temperatures.

The higher nitridation temperature of 1100 °C results in the increased average crystallite sizes D_av_^’^s for both nitride components in the composites (Table 1). Accordingly, in the case of Composite 1, for c-TiN, D_av_ increases from 6 to 23 nm, and for h-AlN, it increases from 5 to 15 nm; whereas in the case of Composite 2, for c-TiN, D_av_ changes from 4 to 13 nm, and for h-AlN, it goes from 3 to 10 nm. Interestingly, there is a significant difference between the composites with Composite 2 showing comparatively smaller-sized crystallites of both components at the two nitridation temperatures. It is worth recalling that Precursor 1, which is used for Composite 1 preparation, is processed via a short room temperature mixing in hexane of the two starting organometallic compounds, whereas in the case of Precursor 2 used for Composite 2, the compounds are subjected to a 3-h reflux in hexane. Since, based on the solution NMR data, there are no obvious chemical interactions between the hexane-mixed organometallic precursors in the Precursors 1 and 2 mixing stages, other factors stemming from the hexane reflux conditions specific to Precursor 2 might have been playing a significant role. In this regard, it is probable that both the monomeric Ti-tetrakis(dimethylamide) and dimeric Al-tris(dimethylamide) are physically bound associates of many alike base species in the solution (the monomeric and dimeric structures, respectively, are resolved in the solid state) which were not differentiated from the chemical environment of the simpler/smaller associates of this type by means of the ^1^H and ^13^C NMR. The somewhat forceful conditions of reflux would then break down those RT-associates into smaller clusters stabilized by interactions with the other amide component. This would be equivalent to better “mixing” of such clusters and smaller metal-specific domains with the resulting smaller average crystallite sizes upon final nitridation. This somewhat unexpected result points to yet another way of nitride powder modification via solvent choice and thermal processing details of the bimetallic precursor solutions before the transamination and nitridation stages.

**Figure 4:**
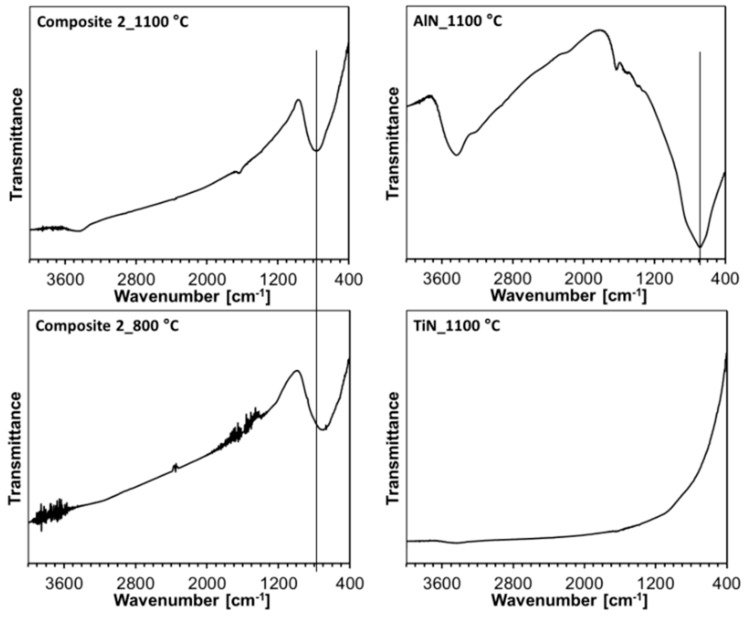
FT-IR spectra of Composite 2 nanopowders from nitridation at 800 and 1100 °C (**left panel**) and individual AlN and TiN nanopowders from nitridation at 1100 °C (**right panel**). Note, non-specific features often include water vapor H_2_O and CO_2_ subtraction bands (noise-like) as well as liquid H_2_O bands (broad features) at ca. 3430 cm^−1^ (stretching mode) and 1640 cm^−1^ (bending mode) for adsorbed water. Solid lines in the range of Al-N stretching vibrations are guides for the eye only.

The infrared spectra for all the powders are similar, and selected examples including Composite 2 are shown in Figure 4. The spectrum for pure aluminum nitride prepared at 1100 °C is characteristic of a rather broad single band centered at ca. 740 cm^−1^ typical for AlN nanopowders [56], whereas the spectrum for titanium nitride, as expected, shows no specific absorption in the mid-infrared range of 400–4000 cm^−1^. For the latter, the apparent curvature of the baseline is due to light scattering and non-specific absorption effects, and, for the former, bands due to some adventitious adsorbed water (3430 and 1640 cm^−1^) are also seen. The spectra for both Composite 2 powders, i.e., from nitridation at 800 and 1100 °C, are characteristic of the single broad band at ca. 730 and 760 cm^−1^, respectively, in the range expected for Al-N stretching vibrations in aluminum nitride. In this regard, the broadness of the peaks and variability in their positions are typical for various nanopowders due to a range of bonding environments in size-distributed AlN nanocrystallites [56]. The FT-IR spectra are consistent with the prevailing Al-N bonding environments, when applicable, and the lack of potential oxidation of the nitrides in both composite and individual component nanopowders (e.g., no strong bands at ca. 600 and 450 cm^−1^ for Al-O stretches and at ca. 500 cm^−1^ for Ti-O stretches [57]).

**Figure 5:**
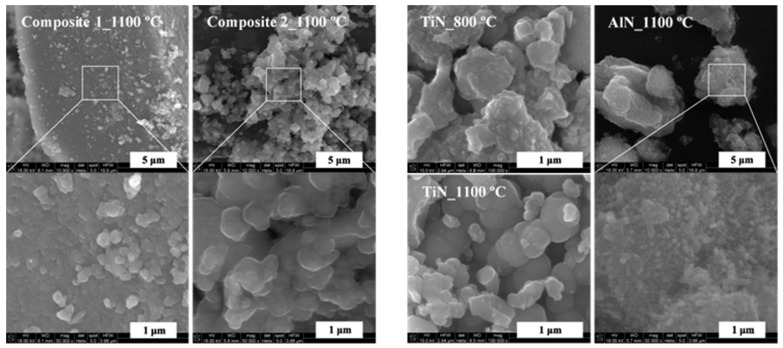
SEM images of selected nanopowders for composite (**left panel**) and individual nitrides (**right panel**). Note that areas in the white-edge rectangles are magnified by factor of 5 and shown in adjoining images.

The scanning electron microscopy examination of the powders confirmed their homogeneity on a submicron scale as demonstrated for the morphologically typical cases of Composites 1 and 2 prepared at 1100 °C (Figure 5). They both appear to be built of blocky aggregates made of smaller, quite uniformly sized submicron particles. These particles are as-if glued, forming a few hundred nanometer large agglomerates that, based on XRD data, contain still smaller nanosized crystallites with the latter too small to be discerned in the SEM images (Table 1, Figure 5, left panel). Those agglomerates seem to be, on average, larger sized for Composite 2. No element mapping was done for the composite nanopowders since, due to varying thickness of the aggregates and the relatively large probing areas of standard EDX mapping, the resolution details below ca. 1 micrometer are not reliably discernible. However, scattered point EDX examination (not shown) reveals that, despite the apparent particle uniformity, there are adjacent areas, with separation of a few micrometers, characteristic of significant variations of one order of magnitude in the relative amounts of Al and Ti. This is consistent with the precursors composed of nanosized domains made of AlN or TiN mixed on the submicron scale, whereas no nitride-specific distinct morphological features are observed. This is not true for the individual nitrides (Figure 5, right panel). On the one hand, the morphology of the TiN powders well reflects the earlier discussed morphology of the derived composites, especially, for the 1100 °C nitridation. On the other hand, the pure AlN prepared at 1100 °C shows relatively long nano-whiskers grown on the surface of micron-sized blocky aggregates. The abundant presence of the whiskers suggests a share of vapor phase crystal growth participating in the formation of bulk AlN from the solid Al-amide-imide precursor. Such high surface area features may have a specific impact on sintering of pure AlN powders. The lack of whiskers in the composite nitrides is therefore significant and points to negligent participation of vapor phase crystallization and to morphological homogeneity.

### 3.2. No-Additive High Pressure and High Temperature (HP-HT) Powder Sintering

The nitride powders were sintered following similar processing as previously reported by us for nanopowders in the system AlN-GaN [49]. This is quite a relevant case since in that report, the similar precursor routes and powder nitridation schemes were also carried out. Specifics are concerned with some differences in nitridation and sintering temperatures. Namely, in this study the nitridation levels were 800 and 1100 °C and sintering temperatures were 650 and 1200 °C, and they were to be compared, respectively, to the previous nitridation at 800 and 950 °C and sintering at 650 and 1000 °C. The lower nitridation temperature of 800 °C was used in both cases, affording powders with a few nanometer average crystallite diameters. The higher temperature level of 950 °C in the AlN-GaN case was dictated by thermal instability of GaN above it. Since both AlN and TiN are reasonably stable, the selection of 1100 °C in this study just yields better crystallized nitrides, compared with the 800 °C-case, which provide a broader spectrum of the nanocrystalite sizes. The selection of sintering temperatures was based on the same two criteria in both studies. First, the lower temperature of 650 °C was below the lower nitridation temperature of 800 °C and, therefore, was anticipated not to contribute to crystal growth during sintering (sintering without temperature-induced recrystallization). And second, the higher temperature of 1200 °C was above the higher nitridation temperature of 1100 °C, which had a possible positive impact on crystal growth during sintering (sintering with temperature-induced recrystallization) while still offering nanocrystalline ceramics. We expected similar effects of pressure here as previously observed. That is, we expected that, at the lower sintering temperature and in the absence of crystal growth, the effects of pressure would cause net crystallite “crushing”, thus lowering average crystallite sizes, and that, at the higher sintering temperature, they would compete with crystal growth. Such a choice of HP-HT sintering conditions in relation to powder nitridation temperatures provided either temperature-active (induced crystallite growth) or pressure-active (induced crystallite crushing) options. In any case, high pressures are essentially used to significantly speed up the sintering of powders.

**Figure 6:**
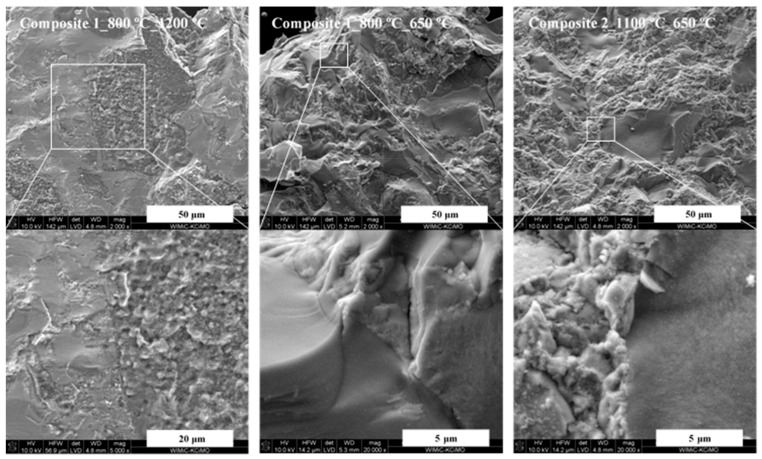
Typical SEM images of fractured composite nanoceramics: (**left**)—Composite 1 made from Precursor 1 nitrided at 800 °C then sintered at 1200 °C; (**middle**)—Composite 1 made from Precursor 1 nitrided at 800 °C then sintered at 650 °C; (**right**)—Composite 2 made from Precursor 2 nitrided at 1100 °C then sintered at 650 °C. Note that areas in white-edge rectangles are magnified and shown in adjoining images.

**Figure 7:**
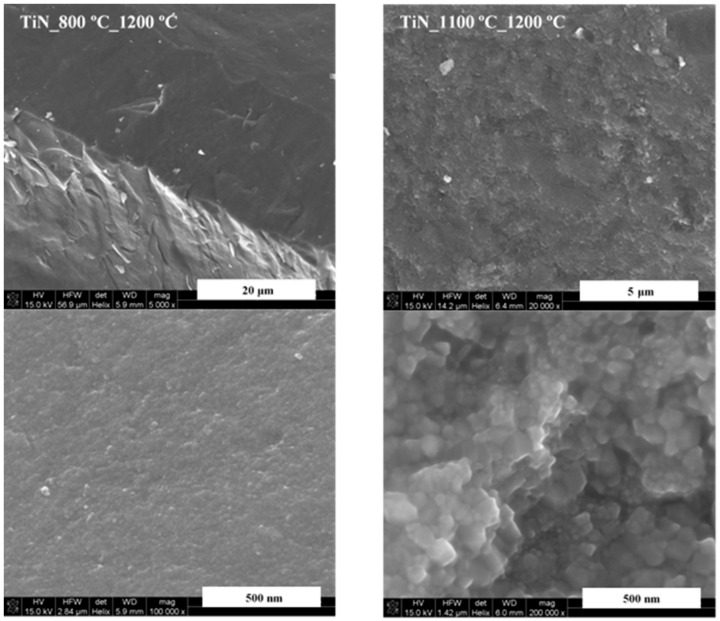
SEM images of pure TiN nanoceramics: (**left**)—powder made at 800 °C and sintered at 1200 °C. Note the solid appearance of fractured area in the left top image; (**right**)—powder made at 1100 °C and sintered at 1200 °C. Note the polished pellet’s surface in the right top image.

Figure 6 shows typical images of intentionally fractured ceramic fragments for selected composite nitride nanoceramics, and Figure 7 presents similar graphics for individual TiN sintering. The original pellets are shown in Figure 1 and are, characteristically, of a golden-dark brown color for the composites and TiN, and graphite-grey color for AlN nanoceramics.

The characteristic feature of all Composite 1 and some Composite 2 ceramics is the presence of two quite well intermixed and relatively large (several tens of micrometer) and distinct domains, each with specific appearance, i.e., solid, dense looking/homogeneous islands with smoothly fractured surfaces as if embedded in an irregularly fractured spiky matrix. It is very likely that this results from various strength field distributions within the compressed microsized domains that show up as different crack-originated features, i.e., highly homogeneous for the flat surfaces and rather variable over shorter distances in the spiky areas. Under the highest magnifications, the solid areas are shown to consist of very similar in size and densely agglomerated crystallites in the several tens of nanometer range. Clearly visible are regular interfaces between the two types of domains (see the higher magnifications). Similar morphology characteristic of uniform nanosized crystallite agglomerates is found for the spiky matrix, too. This is a general feature of all these ceramics independently of whether the sintering temperature supports crystal growth (Figure 6, left) or the temperature is anticipated to be neutral to recrystallization (Figure 6, middle and right) in sintering. However, in the former case the domains become clearly porous with a share of closed microsized pores; apparently, recrystallization phenomena that are associated with intense mass transport/diffusion cause formation of the voids despite the applied compressing high pressure. All this is, generally, also true for fractured fragments of Composite 2 pellets, but the pores appear to be smaller-sized if there are any.

These distinct domains are shown by EDX (elemental point analyses and area mapping) to be practically composed of either AlN in flat smooth-looking fractures and sometimes also with microsized pores or TiN in irregularly fractured spiky areas, as if forming a matrix for the more localized AlN domains. This is a surprising result that suggests an extensive phase/compound segregation towards microsized agglomerates taking place during the relatively short sintering time of 3 min. This has to be confronted with the appearance, as examined by SEM, of the initial composite nanopowders consistent with high particle/nitride homogeneity down to submicron agglomerates with only small-scale but detectable nitride segregation by EDX (Figure 5). There clearly must be a strong driving force during HP-HT sintering for the nitrides segregation and extended aggregation via particle displacement, even without recrystallization and associated mass transport phenomena taking place in specific cases. In this regard, it is conceivable that the two kinds of nitride agglomerates in the starting powders move extensively upon high pressure-induced displacement and, with no affinity for chemical interaction with the counterpart nitride, preferentially bind through contact sites to chemically and crystallographically alike particles to form a specific domain. The visibly increased porosity in the case of Composite 1_800_sint_1200 (Figure 6, left) is likely resultant from intense recrystallization/crystal growth phenomena under the sintering conditions, especially taking place for the extremely low-nanosized crystallites of AlN. With regard to the investigated nitridation vs. sintering conditions with intended recrystallization, the spread between the powder nitridation temperature and sintering temperature is either large (400 °C, i.e., from 800 to 1200 °C) or small (100 °C, i.e., from 1100 to 1200 °C) contributing, among other effects including particle size differences, to various pellet porosities. This should manifest itself in various densities and, possibly, different mechanical hardness in the prepared pool of pellets (vide infra).

The fractured TiN ceramics display homogeneous solid dense surfaces that, upon magnification, are shown to be made of very similar in size but smaller crystallites in the nanosized range (Figure 7). A notable difference in crystallite size can be seen between the 800 °C and 1100 °C-powders sintered at 1200 °C, namely, much bigger and regularly shaped cubic crystallites of varying sizes are encountered in the latter case (Figure. 7, bottom images).

Since the sintering temperature was in both cases higher than the original nitridation temperature (1200 °C vs. 800 and 1100 °C), it also favored some crystal growth with an anticipated higher driving force for the 800 °C-powder due to the higher temperature gradient, i.e., sintering temperature vs. nitridation temperature in this case. However, the sintering time of 3 min is apparently too short for solid state diffusion processes to progress towards equilibrium sizes at 1200 °C for both powders and the initial size-advantage of 57 nm for the 1100 °C-powder vs. 8 nm for the 800 °C-powder (Table 1) is clearly a decisive factor in the net crystal growth in their sintering. The morphology of the pure AlN-derived nanoceramics (not shown) is similar to the TiN’s in that it is also very homogeneous, however, it seems to include more closed microsized pores. Due to the relatively much smaller crystallite sizes, the AlN nanoceramics does not allow for an insightful observation of pellet’s nanocrystallinity by standard SEM as in favorable cases observed for the TiN nanoceramics.

**Figure 8:**
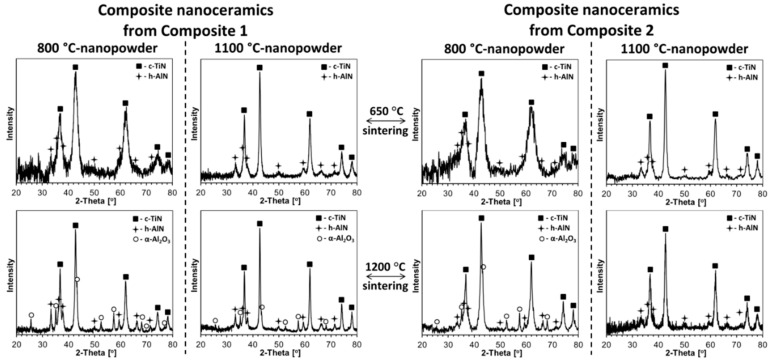
XRD patterns of composite nitride nanoceramics sintered from nanopowders of Composite 1 (**left panel**) and Composite 2 (**right panel**) at 650 and 1200 °C, 7.7 GPa.

**Figure 9:**
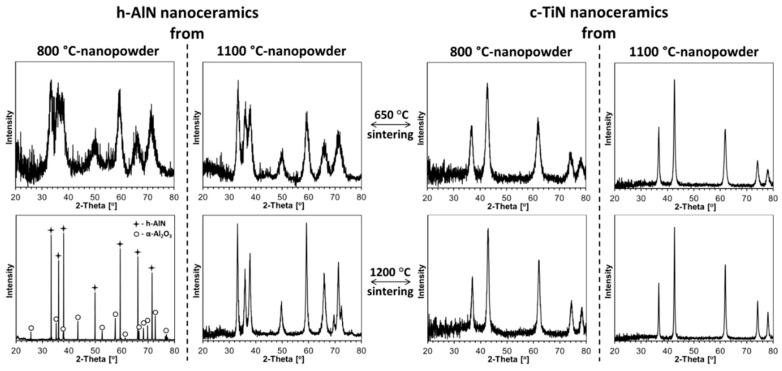
XRD patterns of single nitride nanoceramics sintered from individual nanopowders of h-AlN (**left panel**) and c-TiN (**right panel**) at 650 and 1200 °C, 7.7 GPa. Note some α-Al_2_O_3_ content in bottom corner of left panel for non-standard partially oxidized AlN-800 °C powder then sintered at 1200 °C.

The powder XRD patterns for all ceramics produced from the composite nanopowders are shown in Figure 8, and for reference purposes, the XRD patterns for the individual nitride ceramics of h-AlN and c-TiN are displayed in Figure 9. The evaluated lattice cell constants and average crystallite sizes for all are included in Table 2. In three instances of the composite nanoceramics sintered at 1200 °C, up to several wt.% of unexpected Al_2_O_3_ are present, which points to adventitious air diffusing into the sintering area to oxidize some of AlN but not TiN (Figure 8, the three bottom patterns). In this regard, it is worth knowing that the addition of some Al_2_O_3_ to TiN-AlN is reported to be advantageous for the mechanical strength of such ceramics [58]. A different case of Al_2_O_3_ presence is encountered in the sintering of the 800 °C-powder of presumably pure AlN (Figure 9, left panel, bottom left corner). In this case, the sintering at 650 °C was successfully done immediately upon ampoule opening, but the planned 1200 °C-sintering had to be postponed for 3 weeks due to equipment failure. This resulted in some oxidation of the powder, but it was decided that the sintering should be carried out anyway in the system AlN(85 wt%)-Al_2_O_3_(15 wt%). In the remaining cases, the patterns are satisfactorily assigned to the nitrides with no evidence for formation of significantly doped phases of either c-TiN or h-AlN.

Similarly, as observed by us earlier in the system AlN-GaN [49], the lower sintering temperature of 650 °C (lower than both nitridation temperatures, i.e., sintering without recrystallization) usually results in smaller average crystallite sizes D_av_’s in the nanoceramics than in the starting powders. For extremely small initial D_av_’s, sometimes no change in size is observed. This is illustrated by comparing the sizes D_av_’s (h-AlN/c-TiN) for the Composites 1 and 2 powders (both from 800 and 1100 °C) (Table 1) and 650 °C-derived nanoceramics from them (Table 2): For Composite 1 powders, D_av_ sizes at 800 °C were 5 nm/6 nm and at 1100 °C, 15 nm/23 nm; for 650 °C Composite 1-derived nanoceramics, D_av_ sizes at 800 °C_650 °C were 3 nm/6 nm and at 1100 °C_650 °C, 10 nm/16 nm. Similarly, for Composite 2 powders, D_av_ sizes at 800 °C were 3 nm/4 nm and at 1100 °C, 10 nm/13 nm; and for 650 °C Composite 2-derived nanoceramics, D_av_ sizes at 800 °C_650 °C were 2 nm/4 nm and at 1100 °C_650 °C, 10 nm/13 nm. We assign such a behavior to the “crushing” of nanocrystallites by the extremely high pressure in the absence of crystal growth. However, at the sintering temperature of 1200 °C (sintering with recrystallization) a competition between pressure-resulting crushing and temperature-induced crystal growth yields notably different results, usually with a net crystal growth. And for Composite 1 powders—D_av_ (800 °C: 5 nm/6 nm, 1100 °C: 15 nm/23 nm) and for 1200 °C Composite 1-derived nanoceramics—D_av_ (800 °C_1200 °C: 18 nm/16 nm, 1100 °C_1200 °C: 21 nm/24 nm). Further, for Composite 2 powders—D_av_ (800 °C: 3 nm/4 nm, 1100 °C: 10 nm/13 nm) and for 1200 °C Composite 2-derived nanoceramics—D_av_ (800 °C_1200 °C: 8 nm/8 nm, 1100 °C_1200 °C: 10 nm/13 nm). Interestingly, for both these chemically related composite powders, which show the different average crystallite sizes, the sintering at 1200 °C yields nanoceramics with the range of D_av_’s (h-AlN/c-TiN) from 8 nm/8 nm to 21 nm/24 nm. This range can further be extended by sintering at 650 °C to provide the sizes from 2 nm/4 nm to 10 nm/16 nm. This illustrates benefits of the applied precursor processing routes, which increase the range of structure parameters of the composite nanopowders and resulting nanoceramics.

It is instructive to relate the structure characteristics of the composite nanoceramics to the individual nitride nanoceramics at comparable sintering conditions (Table 1 and Table 2). With exclusion of the non-standard AlN ceramics containing Al_2_O_3_ (Figure 9, left panel, bottom left corner), similar pressure and temperature effects are observed for pure nitrides, as discussed earlier in the composite systems. For pure AlN from 800 °C-nitridation, the powder’s initial D_av_ of 5 nm is unchanged after sintering at 650 °C (no “crushing” size differences). For AlN from 1100 °C-nitridation, the powder’s starting D_av_ of 10 nm can be compared with the calculated 7 nm after 650 °C-sintering and 19 nm after 1200 °C-sintering. The former case is consistent with crystal “crushing”, whereas the latter supports an overall crystal growth winning the competition. These D_av_’s are actually very similar to those found for AlN in the Composite 1-derived nanoceramics, whereas being a bit larger than in the Composite 2 cases. For pure TiN from the 800 and 1100 °C-nitridation, the powders D_av_’s of 8 and 57 nm, respectively, are to be related to sintering at 650 °C with D_av_’s of 8 and 21 nm and sintering at 1200 °C with D_av_’s of 13 and 30 nm. From this comparison, the sintering at 650 °C shows no size change for the 800 °C-powder and the “crushing” effect for the 1100 °C-powder whereas sintering at 1200 °C is diverse in that for the 800 °C-powder the crystal growth predominates and for the 1100 °C-powder crushing is still prevailing. When referred to the D_av_’s of TiN in the composite nanoceramics, the pure TiN-related sizes are consistently larger, supporting a more unrestrained crystal growth conditions in pure TiN or, from another angle, smaller crystallite sizes in the sintered composites with AlN due to the thinning effect of the latter.

The FT-IR spectra for the nanoceramics (not shown) fully confirm bonding characteristics expected for the nitride composites. There is only one broad band peaking in the range 730–750 cm^−1^ that is typical for Al-N bond stretching vibrations in AlN as there are no infrared active bands in the mid-infrared for Ti-N stretches in TiN. These Al-N bands appear to be very similar in shape/symmetry and position when compared with the relevant bands for the initial composite powders.

The Vicker’s hardness and helium density data for the nanoceramics are compiled in Table 3. The Vicker’s hardness, H_v_, was determined by an indentation method under two mass-force loads of 100 and 300 gf (gram-force) yielding comparable results and the data set for 300 gf will be used in discussion. In this regard, too lower load (below ca. 200 gf) indents often display a dependence of hardness on indent depth known as the indentation size effect [59]. In general, the H_v_ values for the sintered pellets are, relatively, high and very high in the range of ca. 7 to 20 GPa. The lowest value of 6.7 GPa is found for nanoceramics made of Composite 2_1100 that is sintered without recrystallization at 650 °C to be compared to the highest of 19.9 GPa for nanoceramics prepared from Composite 1_1100 sintered with recrystallization at 1200 °C. Interestingly, in the former case, it coincides with a noticeable XRD-derived crystallite crushing on sintering, i.e., from D_av_’s for h-AlN/c-TiN of 15 nm/23 nm in the powder to 10 nm/16 nm in the nanoceramics, whereas in the latter case, no significant size changes are observed on sintering, i.e., D_av_’s for h-AlN/c-TiN are found at 10 nm/13 nm both in the powder and in the derived from it nanoceramics. Another case of a high Vicker’s value of 14.9 GPa is for Composite 1_800 sintered also with recrystallization at 1200 °C. In all composite nanoceramics, the Vicker’s hardness is found higher for sintering with recrystallization at 1200 °C compared to the relevant case of the same composite powder sintered at 650 °C when crystallite crushing prevails. This suggests that HP-HT sintering of the composite nitrides with some temperature-induced recrystallization, which overcomes increased particle size variability due to simultaneous pressure-induced crushing, is especially effective in strong particle binding. Such a conclusion also holds true for pure nitride nanoceramics when in each relevant case of sintering at the two temperatures, a significantly higher Vicker’s value is found for the 1200 °C-derived nanoceramics. For the individually sintered nitrides, notably high H_v_’s in the range of 13.5–19.7, GPa are recorded for the c-TiN nanoceramics, whereas only slightly lower H_v_’s ranging from 12.4 to 17.4 GPa are found for the h-AlN nanoceramics. If one relates the Vicker’s hardness of the composite nanoceramics to the hardness of the individual nitride nanoceramics, slightly higher hardness is generally found for the latter ones and the hardness of the h-AlN component (lower than of c-TiN) appears to be a limiting factor in the former.

Finally, these H_v_ values can be referenced to relevant literature data. For single crystalline AlN (vapor deposited thick epitaxial layer), the Vicker’s hardness of 17.7 GPa is reported [60] with theoretical calculations pointing to possible 20 GPa [61]. Moreover, for AlN sintered with various additives, the hardness in the range 10–11 GPa is found [62]. The Vicker’s hardness values of 12.4–17.4 GPa for the sintered pure AlN nanoceramics in this study compares favorably with the data for the single crystalline AlN above. A few micrometer-thick layers of TiN are shown to have hardness of 9.2–15 GPa [63], whereas up to 1 micrometer-thick layers on steel are shown to have hardness of the order of 30 GPa [64]. The range of 13.5–19.7 GPa in this study confirms very good hardness of the bulk TiN nanoceramics. The remarkably high hardness of the metastable cubic Ti_1−x_Al_x_N was mentioned earlier [31,32,33,34,35,36,37]. Although Ti_1–x_A_x_N is not encountered in our ceramics, it is worth quoting its indentation hardness of 24–37 GPa, which is markedly decreased at high temperatures [65]. Somewhat related to the AlN-TiN system are attempts to make mixed metal-metal nitride ceramics as demonstrated by laser melting in the system Ti-AlN yielding composites with Vicker’s hardness of ca. 6.9 GPa [66]. The h-AlN/c-TiN nanoceramics in this study with hardness in the range 6.7–19.9 GPa span from rather good to very high hardness values.

The helium density data provide an additional insight into microstructure of the sintered powders (Table 3). The densities for all sintered composites in the range 2.95–3.47 g/cm^3^ can be compared to 4.22 g/cm^3^, i.e., theoretical value calculated for a 1:1 mixture (on the molar basis) of c-TiN (5.24 g/cm^3^) and h-AlN (3.26 g/cm^3^). It is apparent that the measured densities are rather low to reach 70–82% of this value. For pure AlN nanoceramics (excluding the oxidized sample), these values are in the range 61–72%, whereas for pure nanoceramics of TiN, they are in the range 84–90% when referred to the respective theoretical densities of pure nitrides. The data for the pure nitrides are significant in that they point to generally higher relative porosities of the AlN ceramics compared to the TiN ceramics, while in both cases, supporting quite efficient closed pore formation. Presumably, this is due to more extensive recrystallization processes during sintering of the extremely small nanosized particles of AlN. Therein, the likely course of mass transport events is associated with the disappearance of the smallest and growth of the largest crystallites, as well as with concurrent formation of voids that tend to form closed pores, at least, under non-equilibrium conditions of the 3-min HP-HT sintering. Such phenomena occur also for TiN powders but to a smaller extent. These density results are consistent both with the discussed earlier SEM and XRD data. A pronounced formation of closed microsized pores in AlN domains and extensive nitride segregation/mass transport phenomena are clearly seen in the SEM images for many composite nanoceramics (Figure 6), whereas the pure TiN fractured pellets appear to be much more tightly packed and highly crystallite-size homogeneous (Figure 7). Moreover, the XRD data convincingly support a strong driving force, especially, for AlN crystallites growth and recrystallization. When referring now to the relatively low densities of the composites, they seem to result from the nitrides segregation-crystal growth-pore formation processes, which are kinetically “frozen” due to the short time and diverse mass transport rates for the AlN and TiN components during the HP-HT sintering of their intimately mixed nanopowder composites. Interestingly, there appears to be no clear relationship between the nanoceramics densities and Vicker’s hardness. In this regard, the highest among composites H_v_ values for Composite 1_1100_sint_1200 of 19.9 GPa and for Composite 1_800_1200 of 14.9 GPa are linked, respectively, to the lowest density of 2.95 g/cm^3^ and the second highest density of 3.39 g/cm^3^, whereas the lowest H_v_ for Composite 2_1100_sint_650 of 6.7 GPa corresponds to the relative high density of 3.38 g/cm^3^. Since the helium density data are indicative of abundant closed pores (by SEM, in the microsized range), the latter do not appear to essentially impact the Vicker’s hardness of the nanoceramics.

## 4. Summary and Conclusions

The original two-metal-dimethylamide precursor system enables, via transamination-deamination-nitridation chemistry, the in situ preparation of a range of metal nitride composites of h-AlN and c-TiN synthesis-mixed on a submicron level. The application of different nitridation temperatures of 800 and 1100 °C results in nanocrystalline composites with specific average crystallites sizes of each of the nitrides, characteristic of much smaller sizes of the AlN component. No evidence for the formation of metastable cubic Ti_1-x_Al_x_N is found in any of the preparation options.

The non-additive high pressure (7.7 GPa) and high temperature (650 and 1200 °C) sintering of the composites results in robust bulk nanoceramics in the system TiN-AlN. Similarly, the sintering is applied to the pure individual nitrides to yield equally hard nanoceramics of h-AlN and c-TiN. The sintering process is done either without (650 °C) or with (1200 °C) recrystallization.

The characteristic feature of sintering is phase segregation of the nitrides that, based on SEM examination of fractures, forms quite smoothly shaped AlN domains with sizes up to several tens of micrometer immersed in the irregular looking TiN matrix phase. The phase segregation is accompanied in the 650 °C-sintering by crystallite size deterioration due to high pressure, whereas in the 1200 °C-sintering, it is accompanied by prevailing crystal growth processes. The latter option yields nanoceramics with numerous closed microsized pores, especially in the AlN domains. Mechanical strength of the nanoceramics, as evaluated by a Vicker’s indentation test, ranges from high to very high if compared to the reference values of Vicker’s hardness for the individually sintered TiN and AlN nanopowders and available literature data. The limiting factor in the hardness of the composites often appears to be the relatively lower hardness of the AlN component. In conclusion, the applied sintering conditions with the short sintering time of 3 min result in the kinetically controlled process yielding rather low density/closed microsized pore-containing nanoceramics with surprisingly high mechanical hardness. Further sintering parameter adjustments, especially of pressure and time, are expected to better control the phase segregation and porosity evolution in such bulk TiN-AlN nanoceramics.

## Figures and Tables

**Table 1:** XRD-derived cell parameters and average crystallite sizes determined for composite and individual nitride nanopowders prepared at 800 and 1100 °C in the system TiN-AlN.

Composite Nanopowders	Nitridation Temperature		Individual Nanopowders	Nitridation Temperature	
	800 °C	1100 °C		800 °C	1100 °C
**Composite 1**			**Pure AlN**		
h-AlN:			h-AlN:		
a (Å)	3.10	3.11	a (Å)	3.12	3.12
c (Å)	5.01	4.99	c (Å)	5.02	5.00
D_av_ (nm)	5	15	D_av_ (nm)	5	10
c-TiN:					
a (Å)	4.23	4.24	-	-	-
D_av_ (nm)	6	23			
**Composite 2**			**Pure TiN**		
h-AlN:					
a (Å)	3.08	3.11			
c (Å)	5.02	5.00	-	-	-
D_av_ (nm)	3	10			
c-TiN:			c-TiN:		
a (Å)	4.23	4.24	a (Å)	4.24	4.24
D_av_ (nm)	4	13	D_av_ (nm)	8	57

**Table 2:** XRD-derived crystallographic cell parameters *a* and *c* and average crystallite sizes D_av_’s determined for composite and individual nanoceramics sintered at 650 and 1200 °C, 7.7 GPa in the system TiN-AlN. * Nanoceramics of partially oxidized AlN powder from 800 °C-nitridation and 1200 °C-sintering.

Composite Nanoceramics from	Sintering Temperature		Individual Nanoceramics from	Sintering Temperature	
	650 °C	1200 °C		650 °C	1200 °C
**Composite 1_800**			**Pure AlN_800**		
h-AlN:			h-AlN:		
a (Å)	3.08	3.11	a [Å]	3.12	3.11 *
c (Å)	5.07	4.99	c [Å]	5.08	4.98 *
D_av_ [nm]	3	18	D_av_ [nm]	5	>200 *
c-TiN:			c-TiN:		
a [Å]	4.23	4.24	a [Å]	-	-
D_av_ [nm]	6	16	D_av_ [nm]		
**Composite 1_1100**			**Pure AlN_1100**		
h-AlN:			h-AlN:		
a [Å]	3.11	3.12	a [Å]	3.12	3.12
c [Å]	4.98	4.99	c [Å]	5.00	4.99
D_av_ [nm]	10	21	D_av_ [nm]	7	19
c-TiN:			c-TiN:		
a [Å]	4.25	4.25	a [Å]	-	-
D_av_ [nm]	16	24	D_av_ [nm]		
**Composite 2_800**			**Pure TiN_800**		
h-AlN:					
a [Å]	3.10	3.11			
c [Å]	5.02	5.00	-	-	-
D_av_ [nm]	2	8			
c-TiN:			c-TiN:		
a [Å]	4.22	4.24	a [Å]	4.24	4.24
D_av_ [nm]	4	8	D_av_ [nm]	8	13
**Composite 2_1100**			**Pure TiN_1100**		
h-AlN:					
a [Å]	3.11	3.11			
c [Å]	4.98	4.98	-	-	-
D_av_ [nm]	10	10			
c-TiN:			c-TiN:		
a [Å]	4.24	4.25	a [Å]	4.24	4.24
D_av_ [nm]	13	13	D_av_ [nm]	21	30

**Table 3:** Vicker’s hardness, H_v_ (100 and 300 g-force loads) and helium density, d_He_ data for composite and individual nitride nanoceramics in the system TiN-AlN. Percentages shown in helium density data are calculated with respect to theoretical density of composite c-TiN:h-AlN = 1:1 (molar basis) or of pure nitride. * Nanoceramics of partially oxidized AlN powder.

**Composite Nanoceramics** **from**	Sintering Temperature		**Individual Nanoceramics** **from**	Sintering Temperature	
	650 °C	1200 °C		650 °C	1200 °C
**Composite 1_800**			**Pure AlN_800**		
d_He_ (SD) [g/cm^3^]	3.25 (0.05)	3.39 (0.02)	d_He_ (SD) [g/cm^3^]	2.27 (0.03)	3.32 *
theor	77%	80%	theor	70%	102%
H_V_ (SD) [GPa]:			H_V_ (SD) [GPa]:		
under 100 gf	13.1 (2.0)	12.1 (2.2)	under 100 gf	13.8 (0.6)	n/d
**under 300 gf**	**13.1 (1.0)**	**14.9 (3.6)**	**under 300 gf**	**12.7 (1.1)**	
**Composite 1_1100**			**Pure AlN_1100**		
d_He_ (SD) [g/cm^3^]	3.33 (0.03)	2.95 (0.04)	d_He_ (SD) [g/cm^3^]	2.34 (0.02)	2.00 (0.03)
theor	79%	70%	theor	72%	61%
H_V_ (SD) [GPa]:			H_V_ (SD) [GPa]:		
under 100 gf	9.6 (1.9)	21.9 (2.9)	under 100 gf	13.9 (0.9)	18.9 (1.1)
**under 300 gf**	**10.8 (1.7)**	**19.9 (3.5)**	**under 300 gf**	**12.4 (0.8)**	**17.4 (0.8)**
**Composite 2_800**			**Pure TiN_800**		
d_He_ (SD) [g/cm^3^]	3.23 (0.02)	2.99 (0.03)	d_He_ (SD) [g/cm^3^]	4.51 (0.06)	4.39 (0.04)
theor	77%	71%	theor	86%	84%
H_V_ (SD) [GPa]:			H_V_ (SD) [GPa]:		
under 100 gf	11.0 (1.2)	13.4 (1.1)	under 100 gf	15.4 (1.6)	20.5 (1.7)
**under 300 gf**	**11.2 (1.3)**	**12.2 (1.5)**	**under 300 gf**	**15.5 (0.8)**	**19.7 (1.6)**
**Composite 2_1100**			**Pure TiN_1100**		
d_He_ (SD) [g/cm^3^]	3.38 (0.04)	3.47 (0.03)	d_He_ (SD) [g/cm^3^]	4.59 (0.06)	4.72 (0.05)
theor	80%	82%	theor	88%	90%
H_V_ (SD) [GPa]:			H_V_ (SD) [GPa]:		
under 100 gf	7.3 (2.0)	14.8 (0.8)	under 100 gf	14.3 (1.0)	18.8 (2.4)
**under 300 gf**	**6.7 (1.3)**	**11.9 (3.7)**	**under 300 gf**	**13.5 (0.9)**	**17.7 (2.2)**

## Data Availability

The data presented in this study are available on request from the corresponding author.

## References

[1] Ashraf I., Rizwan S., Iqbal M. (2020). A comprehensive review on the synthesis and energy applications of nano-structured metal nitrides. Front. Mater..

[2] Dongil A.B. (2019). Recent progress on transition metal nitrides nanoparticles as heterogeneous catalysts. Nanomaterials.

[3] Jena D., Page R., Casamento J., Dang P., Singhal J., Zhang Z., Wright J., Khalsa G., Cho Y., Xing H.G. (2019). The new nitrides: Layered, ferroelectric, magnetic, metallic and superconducting nitrides to boost the GaN photonics and electronics eco-system. Jpn. J. Appl. Phys..

[4] Zakutayev A. (2016). Design of nitride semiconductors for solar energy conversion. J. Mater. Chem. A.

[5] Zhang Z., Chai C., Zhang W., Song Y., Kong L., Yang Y. (2020). First-principles study on III-nitride polymorphs: AlN/GaN/InN in the Pmn2_1_ phase. Materials.

[6] Watson I.M. (2013). Metal organic vapour phase epitaxy of AlN, GaN, InN and their alloys: A key chemical technology for advanced device applications. Coordin. Chem. Rev..

[7] Van Schilfgaarde M., Sher A., Chen A.B. (1997). Theory of AlN, GaN, InN and their alloys. J. Cryst. Growth.

[8] Tsareva A.M., Leonov A.V., Lysenkov A.S., Sevostyanov M.A. (2019). Methods of producing ceramic on the basis of metal nitrides (Review). Glass Ceram..

[9] Chernyavskii A.S. (2019). Synthesis of ceramics based on titanium, zirconium, and hafnium nitrides. Inorg. Mater..

[10] Tareen A.K., Priyanga G.S., Behara S., Thomas T., Yang M.H. (2019). Mixed ternary transition metal nitrides: A comprehensive review of synthesis, electronic structure, and properties of engineering relevance. Prog. Solid State Chem..

[11] Höhn P., Niewa R. (2017). Nitrides of non-main group elements. Handb. Solid State Chem..

[12] Sun W.H., Bartel C.J., Arca E., Bauers S.R., Matthews B., Orvananos B., Chen B.R., Toney M.F., Schelhas L.T., Tumas W. (2019). A map of the inorganic ternary metal nitrides. Nature Mater..

[13] Levason B., Hector A.L. (2013). Chemistry and applications of metal nitrides. Coordin. Chem. Rev..

[14] Iwata A., Akedo J. (2005). Hexagonal to cubic crystal structure transformation during aerosol deposition of aluminum nitride. J. Cryst. Growth.

[15] Nersisyan H.H., Yoo B.U., Lee K.H., Lee J.H. (2015). A thermochemical pathway for controlled synthesis of AlN nanoparticles in non-isothermal conditions. Thermochim. Acta.

[16] Sung M.C., Wang Y.F., Chen S.C., Tsai C.H. (2019). Two-stage plasma thermal nitridation processes for the production of aluminum nitride powders from aluminum powders. Materials.

[17] Lei W.W., Liu D., Zhang J., Liu B.B., Zhu P.W., Cui T., Cui Q.L., Zou G.T. (2009). AlN nanostructures: Tunable architectures and optical properties. Chem. Commun..

[18] Janik J.F., Wells R.L., Coffer J.L., St. John J.V., Pennington W.T., Schimek G.L. (1998). Nanocrystalline aluminum nitride and aluminum/gallium nitride nanocomposites via transamination of [M(NMe_2_)_3_]_2_, M = Al, Al/Ga(1/1). Chem. Mater..

[19] Patsalas P., Kalfagiannis N., Kassavetis S. (2015). Optical properties and plasmonic performance of titanium nitride. Materials.

[20] Wang L., Jiang W., Chen L., Yang M., Zhu H. (2006). Consolidation of nano-sized TiN powders by spark plasma sintering. J. Am. Ceram. Soc..

[21] Lengauer W. (1992). Properties of bulk δ-TiN_1−x_ prepared by nitrogen diffusion into titanium metal. J. Alloys Compd..

[22] Giardini A., Marotta V., Orlando S., Parisi G.P. (2002). Titanium nitride thin films deposited by reactive pulsed-laser ablation in RF plasma. Surf. Coat. Tech..

[23] Cheng H.E., Wen Y.W. (2004). Correlation between process parameters, microstructure and hardness of titanium nitride films by chemical vapor deposition. Surf. Coat. Technol..

[24] Gea W.Y., Changa Z., Siddiquea A., Shia B., Liu C. (2020). Large-area fabrication of TiN thin films with photothermal effect via PECVD. Ceram. Int..

[25] Liu Y.J., Wang Y., Zhang Y., You Z.X., Lv X.W. (2020). Mechanism on reduction and nitridation of micrometer-sized titania with ammonia gas. J. Am. Ceram. Soc..

[26] Alhussain H., Mise T., Matsuo Y., Kiyono H., Nishikiori K., Akashi T. (2019). Influence of ammonia gas exposure on microstructure of nanocrystalline titanium nitride powder synthesized from titanium dioxide. J. Ceram. Soc. Jpn..

[27] Drygas M., Czosnek C., Paine R.T., Janik J.F. (2006). Two-stage aerosol synthesis of titanium nitride TiN and titanium oxynitride TiO_x_N_y_ nanopowders of spherical particle morphology. Chem. Mater..

[28] Schuster J.C., Bauer J. (1984). The ternary system titanium-aluminum-nitrogen. J. Solid State Chem..

[29] Chen Q., Sundman B. (1998). Thermodynamic assessment of the Ti-AI-N system. J. Phase Equilib..

[30] Han Y.S., Kalmykov K.B., Dunaev S.F., Zaitsev A.I. (2004). Solid-state phase equilibria in the titanium-aluminum-nitrogen system. J. Phase Equilib. Diff..

[31] Endler I., Hohn M., Herrmann M., Pitonak R., Ruppi S., Schneider M., van den Berg H., Westphal H. (2008). Novel aluminum-rich Ti_1−x_Al_x_N coatings by LPCVD. Surf. Coat. Technol..

[32] Waters C.K., Yarmolenko S., Sankar J., Neralla S., Kelkar A.D. (2005). Synthesis, optimization, and characterization of AlN/TiN thin film heterostructures. Nanoengineering of Structural, Functional and Smart Materials.

[33] Uny F., Blanquet E., Schuster F., Sanchette F. (2018). Ti-Al-N-based hard coatings: Thermodynamical background, CVD deposition, and properties. A review. Coatings and Thin Film Technologies.

[34] Liu S., Chang K., Mraz S., Chen X., Hans M., Music D., Primetzhofer D., Schneider J.M. (2019). Modeling of metastable phase formation for sputtered Ti_1-x_Al_x_N thin films. Acta Mater..

[35] Krylov I., Qi Y.S., Korchnoy V., Weinfeld K., Eizenberg M., Yalon E. (2020). Zero temperature coefficient of resistance in back-end-of-the-line compatible titanium aluminum nitride films by atomic layer deposition. Appl. Phys. Lett..

[36] Rogstrom L., Ullbrand J., Almer J., Hultman L., Jansson B., Oden M. (2012). Strain evolution during spinodal decomposition of TiAlN thin films. Thin Solid Films.

[37] Shimizu T., Teranishi Y., Morikawa K., Komiya H., Watanabe T., Nagasaka H., Yang M. (2015). Impact of pulse duration in high power impulse magnetron sputtering on the low-temperature growth of wurtzite phase (Ti,Al)N films with high hardness. Thin Solid Films.

[38] Du R.X., Okamura H., Watanabe R., Kawasaki A. (2004). Characterization of TiN-AlN composites prepared through mechanical alloying and followed by pressure sintering. Mater. Trans..

[39] Mosina T.V. (2014). Contact strength, crack resistance, and abrasive wear of composite materials belonging to the system TiN-AlN. Refract. Ind. Ceram..

[40] Mosina T.V. (2014). Electric-spark alloying of composite material of the systems TiN-AlN and TiN-AlN-(Ni-Cr) as a method for applying wear-resistant coatings. Refract. Ind. Ceram..

[41] Kim W., Lim J.W., Oh H.S., Shon I.J. (2014). Mechanical properties of nanostructured TiN-AlN composites rapidly consolidated by pulsed current activated sintering. Ceram. Int..

[42] Zgalat-Lozinskii O.B. (2015). Structure of Si_3_N_4_-Y_2_O_3_-Al_2_O_3_ and TiN-AlN composites consolidated in microwaves (2.45 GHz). Powder Metall. Met. Ceram..

[43] Lavrenko V.A., Shvets V.A., Talas V.N. (2015). Corrosion of AlN-TiN ceramics in 3% NaCl solution. Powder Metall. Met. Ceram..

[44] Ordanyan S.S., Krylov S.O., Stepanenko E.K., Tsielin U.A. (1995). Some special features of structure formation in sintering ultradisperse powders of TiN-AlN compositions. Refractories.

[45] Radajewski M., Henschel S., Grutzner S., Kruger L., Schimpf C., Chmelik D., Rafaja D. (2016). Microstructure and mechanical properties of bulk TiN-AlN composites processed by FAST/SPS. Ceram. Int..

[46] Radune M., Zinigrad M., Kalabukhov S., Sokol M., Chumanov V.I., Frage N. (2016). Spark plasma sintering of Ti_1-x_Al_x_N nano-powders synthesized by high-energy ball milling. Ceram. Int..

[47] He Z.W., Wang M.Z., Xu S., Zhao Y.C., Zou Q. (2017). Reactive formation of AlN precipitates and epitaxial interface in TiN_1-x_-AlN composites. J. Ceram. Soc. Jpn..

[48] Kudyakova V.S., Chukin A.V., Dorokhin M.V., Kuznetsov Y.M., Shishkin R.A., Beketov A.R. (2019). Structure, microhardness and thermal conducting properties of the high-pressure high-temperature-treated Al-Ti-N materials. Appl. Phys. A.

[49] Drygas M., Kapusta K., Janik J.F., Bucko M.M., Gierlotka S., Stelmakh S., Palosz B., Olejniczak C. (2020). Novel nanoceramics from in situ made nanocrystalline powders of pure nitrides and their composites in the system aluminum nitride AlN/gallium nitride GaN/aluminum gallium nitride Al_0.5_Ga_0.5_N. J. Eur. Ceram. Soc..

[50] Brown G.M., Maya L. (1988). Ammonolysis products of the dialkylamides of titanium, zirconium, and niobium as precursors to metal nitrides. J. Am. Ceram. Soc..

[51] Stelmakh S., Grzanka E., Gierlotka S., Janik J.F., Drygas M., Lathe C., Palosz B. (2011). Compression and thermal expansion of nanocrystalline TiN. Z. Kristallogr. Proc..

[52] Maya L. (1986). Synthetic approaches to aluminum nitride via pyrolysis of a precursor. Adv. Ceram. Mater..

[53] Borysiuk J., Caban P., Strupinski W., Gierlotka S., Stelmakh S., Janik J.F. (2007). TEM investigations of GaN layers grown on silicon and sintered GaN nano-ceramic substrates. Cryst. Res. Technol..

[54] Janik J.F., Drygas M., Czosnek C., Pałosz B., Gierlotka S., Stelmakh S., Grzanka E., Kalisz G., Swiderska-Sroda A., Leszczynski M. (2012). Sposób Wytwarzania Spieków Azotku Galu GaN (in Polish)-Way to Make Sintered Gallium Nitride GaN. Polish Patent.

[55] Bradley D.C., Gitlitz. M.H. (1969). Metallo-organic compounds containing metal-nitrogen bonds. Part VI. Infrared and nuclear magnetic resonance of dialkylamido-derivatives of titanium. J. Chem. Soc. A.

[56] Bellucci S., Balasubramanian C., Cinque G., Marcelli A., Cestelli Guidi M., Piccinini M., Popov A., Soldatov A., Onorato P. (2006). Characterization of aluminium nitride nanostructures by XANES and FTIR spectroscopies with synchrotron radiation. J. Phys. Condens. Matter.

[57] Choi D., Kumta P.A. (2006). Nanocrystalline TiN derived by a two-step halide approach for electrochemical capacitors. J. Electrochem. Soc..

[58] Liles K.J. (1989). Mechanical and Physical Properties of Particulate Composites in the System Titanium Nitride-Alumina-Aluminum Nitride.

[59] Pharr G.M., Herbert E.G., Gao Y.F. (2010). The indentation size effect: A critical examination of experimental observations and mechanistic interpretations. Annu. Rev. Mater. Res..

[60] Yonenaga I. (2001). Mechanical stability of power device materials, high temperature hardness of SiC, AlN and GaN. Chem. Sustain. Dev..

[61] Kishore N., Nagarajan V., Chandiramouli R. (2019). Mechanical and electronic properties under high pressure on ternary AlGaN and InGaN compound—A first-principles perspective. Mater. Res. Express.

[62] Xiang M., Zhou Y.F., Xu W.T., Li X.Q., Wang K., Pan W. (2018). Transparent AlN ceramics sintered from nanopowders produced by the wet chemical method. J. Ceram. Soc. Jpn..

[63] Kuo C.C., Lin Y.T., Chan A., Chang J.T. (2019). High temperature wear behavior of titanium nitride coating deposited using high power impulse magnetron sputtering. Coatings.

[64] Stone D.S., Yoder K.B., Sproul W.D. (1991). Hardness and elastic modulus of TiN based on continuous indentation technique and new correlation. J. Vac. Sci. Technol. A.

[65] Bartosik M., Rumeau C., Hahn R., Zhang Z.L., Mayrhofer P.H. (2017). Fracture toughness and structural evolution in the TiAlN system upon annealing. Sci. Rep..

[66] Sitek R., Szustecki M., Zrodowski L., Wysocki B., Jaroszewicz J., Wisniewski P., Mizer J. (2020). Analysis of microstructure and properties of a Ti–AlN composite produced by selective laser melting. Materials.

